# Digitization of Historical Data from Somalia’s Last Smallpox Outbreaks 1976-1977

**DOI:** 10.1038/s41597-026-07343-8

**Published:** 2026-05-14

**Authors:** Rabia Khan, Hannah Williams, Galena Kuyumdzhieva, Christopher Carroll, Katherine Griffiths, Leonardo Gada, Brodie Walker, Joseph Shingleton, Joe Flannagan, Rosamund Lewis, Thomas Finnie, Ian Hall, Emma Bennett

**Affiliations:** 1 Epidemiological Insight Team, Analysis and Intelligence Assessment, Chief Data Officer Group, UK Health Security Agency, London, UK; 2 Defence Science and Technology Laboratory, Salisbury, UK; 3 Advanced Analytics, Analysis and Intelligence Assessment, Chief Data Officer Group, UK Health Security Agency, London, UK; 4 School of Biological Sciences, University of Manchester, Manchester, UK; 5 Modelling and Data Science for Emergency Preparedness, Resilience and Response, Analysis and Intelligence Assessment, Chief Data Officer Group, UK Health Security Agency, London, UK; 6 School of Health and Social Wellbeing, University of West of England, Bristol, UK; 7 School of Geographical and Earth Sciences, University of Glasgow, Glasgow, UK; 8 Mathematical Epidemiology Group, Department of Mathematics, University of Manchester, Manchester, UK; 9 Smallpox Secretariat, High Threat Pathogens, Epidemic and Pandemic Management Department, Health Emergencies Programme, World Health Organization, Geneva, Switzerland; 10 Christabel Pankhurst Institute, University of Manchester, Manchester, United Kingdom

**Keywords:** Infectious diseases, Data publication and archiving

## Abstract

Over more than three thousand years, smallpox caused millions of deaths worldwide. With smallpox declared eradicated in 1980, potential use of the variola virus as a bioterrorist threat remains a concern. While modern disease outbreaks are modelled using current data, understanding how to control an eradicated disease such as smallpox relies on the analysis of historical data. Handwritten smallpox outbreak data from Somalia (1976–1977) were obtained from the World Health Organization by a Public Health England study team (now UK Health Security Agency). The data were manually transcribed into a Microsoft Excel worksheet and uploaded to the UK Data Service ReShare repository. The transcribed line list comprises of 3,255 incident cases and includes the following variables: national case serial number, age, sex, date of rash onset, date detected, village/locality, district, region, regional outbreak number, and national outbreak number. The aim of this study is to make these historical data globally accessible, enhancing our understanding of smallpox transmission in Somalia during the disease eradication period and drawing lessons for future outbreaks.

## Background & Summary

Smallpox is caused by the variola virus, which belongs to the *Orthopox* virus genus, a member of the *Poxviridae* family^1^. During the 1950s, an estimated 50 million cases occurred globally each year^1^. The virus itself consists of two strains, *variola minor* with a mortality rate typically between 0.4–1.5% and *variola major*, the more severe and virulent form, with a mortality rate typically between 16–25% in unvaccinated populations^1^.

The smallpox virus is primarily transmitted through aerosolised respiratory droplets caused by lesions in the oropharynx releasing virus into the saliva^1^ and can also transmit via fomites and direct contact with cutaneous lesions^1^. The incubation period for both the strains is usually 10–14 days, but in rare cases it can range from 7 to 19 days^2^. *Variola minor* generally causes fewer and less severe symptoms than *variola major*^1,3^. Symptoms for this strain include fever, headache, occasionally backache, and the appearance of a rash beginning on the face and extremities^1^. Residual scarring and pockmarks are less common than in *variola major*^1,2^.

Following the formal initiation of the World Health Organization (WHO)’s Smallpox Eradication Programme in 1967, Somalia launched its national eradication programme in 1969, implementing population-wide vaccination with a target coverage of at least 80% to achieve herd immunity. However, logistical challenges including civil unrest, remote and hard-to-reach regions, and nomadic populations made achieving uniform coverage difficult in some areas^1^.

Between 1975 and 1977, the national strategy shifted from mass vaccination to a more targeted surveillance and containment approach^1,4^. In September 1976, after 14 years of apparent absence, endemic smallpox cases re-emerged in Somalia^1^. The first confirmed cases in Somalia had rash onset on 30 August and 5 September 1976^4^. Laboratory confirmation of smallpox in two patients in Mogadishu on 27 September 1976 was followed shortly thereafter by three additional cases; despite intensive investigations along the Ogaden border, the source of transmission was never established^4^.

Locally, Somali teams, aided by WHO advisers, immediately concentrated on border searches, assuming importation of smallpox cases from Ethiopia, while WHO rapidly intensified surveillance, field investigations, containment measures, and international coordination^4^.

Furthermore, local and WHO field staff increased sharply from 381 in March to 3,301 in June 1977, peaking as part of the emergency response to the rising number of cases.

Suspected cases were identified through active community searches for individuals presenting with rash-and-fever, as well as through reports from local healthcare workers and notifications from the public. These cases were then verified through clinical assessment with additional cases identified via contact tracing.

Additionally, in 1977, standardized specimen collection procedures were introduced in Somalia, under WHO guidance^4^. Mandatory specimen collection was required for clinically diagnosed (from August 1977) and suspected smallpox and chickenpox cases, particularly in unvaccinated individuals; rash-with-fever cases resulting in death, cases under containment measures (from October 1977), diagnostically uncertain rash-with-fever cases, and cases suspected of post-vaccination smallpox^4^.

In February 1977, a cash reward scheme was introduced in Somalia to encourage public reporting of suspected smallpox cases within six weeks of rash onset. Payment was made only after laboratory confirmation of smallpox and was restricted to the first case identified within an outbreak^4^. A dual-incentive structure provided 200 Somali shillings to both the initial informant and the verifying health worker, thereby strengthening surveillance capacity. Although this system enhanced case detection overall, its impact was more limited among highly mobile nomadic populations^4^.

Smallpox was officially declared eradicated at the World Health Assembly in 1980^5^. The last naturally occurring case of *variola major* was reported in Bangladesh in October 1975, and the final known case of *variola minor* occurred in Somalia, in October 1977^1,6^. The eradication of the disease implies that contemporary populations lack immunity and may therefore be vulnerable to deliberate release of the smallpox virus through bioterrorism. This continues to generate debate around the most appropriate modelling strategies for planning public health interventions in non-immune populations^7,8^. Whereas the dynamics of disease outbreaks in the 21st century, and the outcomes of control strategies used to contain them, have been predicted via models parameterised with contemporary outbreak data (e.g., measles immunisation campaigns), to understand how an eradicated disease, such as smallpox, might be controlled, requires an analysis of historical outbreak data.

The outbreak data described in this study were originally collected as part of the World Health Organization’s smallpox eradication activities in Somalia and were later provided by the WHO to Public Health England’s (now UK Health Security Agency) Emergency Response Department. This study describes the manual transcription or the original handwritten documents, and the cleaning, and validation of the transcribed dataset. The resulting dataset has been made publicly available to support research by the global scientific community.

This transcribed dataset can be used for descriptive analysis of the last global smallpox outbreaks in Somalia, including the construction of epidemic curves, statistical modelling, regression analysis and development of compartmental infectious disease models. In combination with Somalia’s census data, the line list can enable creation of pseudo-populations for simulation studies and retrospective outbreak reconstruction.

Since the last naturally occurring case of *variola major* was reported in 1975, prior to the 1976–1977 outbreak in Somalia, the cases recorded in this dataset are assumed to represent *variola minor*.

## Methods

Due to challenges with the quality of the original scanned document and indistinct handwriting in some records, a common issue in projects involving handwritten documents, the PDF was unsuitable for automated text extraction and therefore required manual data transcription.

**Fig. 1:**
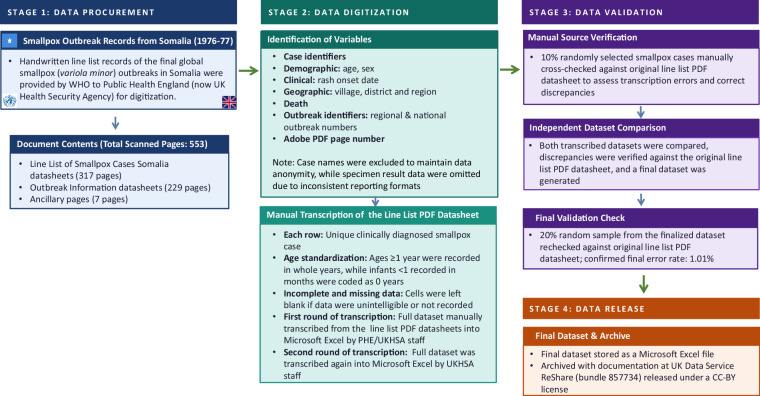
Flowchart illustrating the workflow for procuring, digitizing and processing historical World Health Organization’s smallpox outbreak records from Somalia (1976–1977).

A summary of the data procurement and transcription workflow is presented in Fig. 1.

### Ethics approval

The authors did not interact with human participants or collect primary data. Instead, this work involved the transcription of an existing archival dataset for public dissemination provided by WHO.

The transcribed dataset shared in this study is fully anonymised and does not contain personally identifiable information. Ethical approval from UKHSA, WHO, the University of Manchester, or a third-party institutional review board was therefore not required for this work.

### Description of the original PDF line list document

The outbreak data from Somalia were originally collected on two types of datasheets entitled: 1) *Line List of Smallpox Cases Somalia* and 2) *Outbreak Information*. The data entry for this dataset was based primarily on the *Line list of Smallpox Cases in Somalia* datasheets and supplemented with data from the *Outbreak Information* datasheets where relevant. All datasheets were contained within a single scanned PDF document comprising 553 pages (Table 1). Of these, 317 pages consisted of *Line List of Smallpox Cases Somalia* datasheets, 229 pages contained *Outbreak Information* datasheets, and the remaining 7 pages were non-data administrative pages.

**Table 1:** Summary of page types in the World Health Organization’s original smallpox outbreak line list document.

WHO line list PDF page range (original document)	Number of pages containing the *Line List of Smallpox Cases Somalia* datasheets	Number of pages containing the *Outbreak Information* datasheets	Other pages	Total pages
1–50	48	0	2	50
51–100	50	0	0	50
101–150	50	0	0	50
151–200	50	0	0	50
201–250	28	19	3	50
251–300	6	43	1	50
301–350	1	49	0	50
351–400	42	7	1	50
401–450	40	10	0	50
451–500	0	50	0	50
501–553	2	51	0	53
Total	317	229	7	553

Data from the *Line List of Smallpox Cases Somalia* datasheets and *Outbreak Information* datasheets were transcribed. Pages classified as “Other” include blank pages or ancillary material and were not included in the transcription.

The PDF of the original line list document can be obtained from the WHO by contacting the Smallpox Secretariat at smallpoxsecretariat@who.int.

### The line list of smallpox cases somalia datasheets

**Fig. 2:**
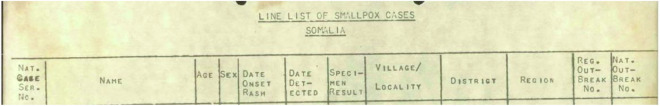
Screenshot of the “Line List of Smallpox Cases Somalia” datasheet, World Health Organization 1976–1977.

The *Line List of Smallpox Cases Somalia* datasheets consisted of the following variables: *National Case Serial Number, Name, Age, Sex, Date Onset Rash, Specimen Result, Date Detected, Village/ Locality, District, Region, Regional Outbreak Number and National Outbreak Number* (Fig. 2).

Cases recorded in the line list represent smallpox cases identified as part of the smallpox surveillance. Case identification during the surveillance relied primarily on clinical diagnosis based on the WHO smallpox case definition, which included a febrile prodrome followed by the characteristic progression and distribution of the distinct smallpox rash^4^. Trained surveillance staff and WHO field investigators used these criteria during case-finding activities to rapidly identify cases and initiate containment measures such as isolation, contact tracing, and vaccination.

According to WHO reports on the Somalia eradication programme, a total of 879 specimens of crusts and vesicular fluid were collected in 1977, of which 265 (30.14%) tested positive for variola virus^4^. However, laboratory capacity was limited and laboratory testing was used primarily for surveillance validation and differential diagnosis (e.g., distinguishing smallpox from chickenpox) rather than as a routine diagnostic requirement for all cases. Hence not all clinically diagnosed cases presented in this dataset were laboratory confirmed.

In this transcribed dataset, the records represent all cases documented in the original line lists, including individuals clinically diagnosed with smallpox and those for whom specimen collection was performed. However, specimen test results were not included in the original line list documents and were not available to the authors; therefore, they are not included in the transcribed dataset.

### The outbreak information datasheets

**Fig. 3:**
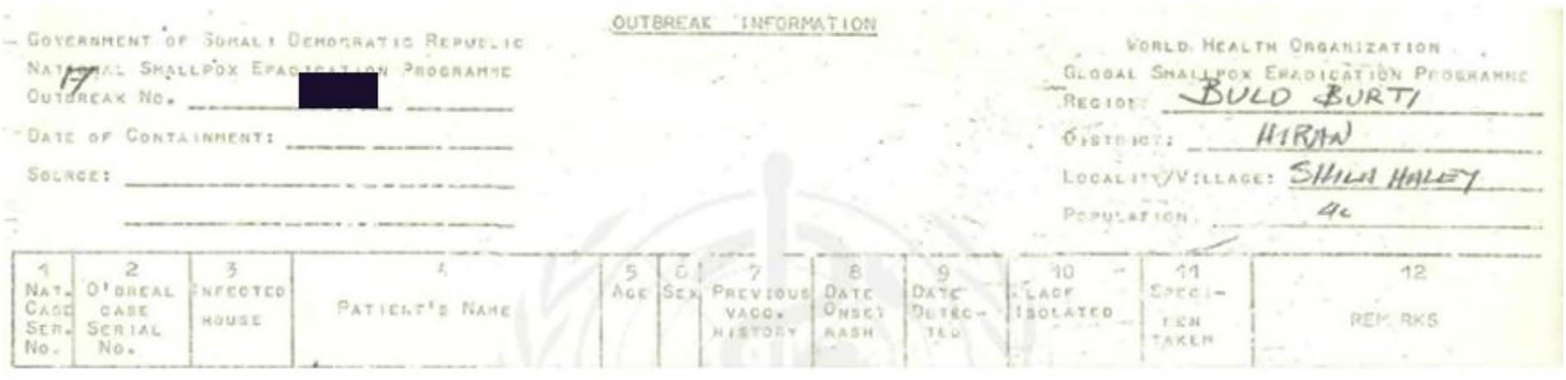
Screenshot of the “Outbreak Information” datasheet, World Health Organization 1976–1977.

The *Outbreak Information* datasheets included the following data columns: *Outbreak Number, Date of Containment, Source, Region, District, Locality/Village, Population, National Case Serial number, Outbreak Case Serial number, Infected House, Patient’s Name, Age, Sex, Previous Vaccination History, Date Onset Rash, Date Detected, Place Isolated (i.e. the place where the patient was isolated), Specimen Taken, and Remarks* (Fig. 3). The outbreak information sheets included details for a subset of the cases only.

While the Outbreak Information datasheets did not introduce new data, they were useful for cross-referencing data including clarifying ambiguous or missing data from the line list datasheets for the 578 cases. Where inconsistencies arose in case information between the *Line List of Smallpox Cases Somalia* and *Outbreak Information* datasheets, inference was made as far as possible using neighbouring cases and line list sheets. Where an individual did not have a corresponding *Outbreak Information* datasheet, the data from the *Line List of Smallpox Cases Somalia* datasheet were considered the authoritative source.

### Data transcription process

Since the scanned PDF document contained personally identifiable information, both the original file and the Microsoft Excel data entry file were stored in a password protected folder. Access was restricted to team members directly involved in the study or the transcription process. All team members completed the Security and Data Protection training provided by Public Health England prior to commencing the data transcription.

The digitization process involved two independent rounds of manual transcription of the PDF document into Microsoft Excel files. The first transcription was conducted by five team members (HW, BW, JS, DM, MG) and the second by two team members (CC and GK).

Data validation constraints were applied on the Microsoft Excel cells to ensure the data transcription adhered to good practice. Informative warnings were displayed if a data entry did not meet the specified conditions for a given variable. The variable names, data type and allowed values are summarised in Table 2.

**Table 2:** Data variable definitions and allowed values for the transcribed smallpox line list dataset (1976–1977).

Variable name in the transcribed Excel datasheet	Data type	Allowed values	Description
Ã¯..Nat.case.ser.no	Integer	≥1 (unique identifier)	National case serial number assigned to each smallpox case in the line list. Each record contains a unique value.
Age	Integer	0–100; blank if missing	Age of the individual in years. Ages were transcribed as whole numbers. Ages recorded as months for infants less than 1 year of age in the original record were transcribed as 0.
Sex	Text (categorical)	m, f, blank	Sex of the individual: m = male, f = female; blank indicates missing or not recorded.
Date.onset.rash	Date	dd/mm/yyyy; blank	Date of rash onset as recorded in the original line list. Represents the reported start of smallpox rash symptoms. Blank indicates missing or not recorded.
Date.detected	Date	dd/mm/yyyy; blank	Date the case was detected or reported through the surveillance system. Blank indicates missing or not recorded.
Village	Text	Free text; 1,028 unique values*	Village or locality with the reported case.
District	Text (categorical)	37 unique district names: abudwak, adala, adenyabal, afgoi, baidoa, balad, bardere, boale, bondhere, bulo burti, bulo hawa, bur akaba, burao, dinsor, el barde, el bur, el wak, garbahare, gelib, gilia, hodan, huddum, jalalaksi, jamame, jowhar, kansadere, kismayo, koryole, kurtunwarey, luk, merca, saakow, teyeglow, wadajir, wajid, wanlewein, yet	District associated with the reported case.
Region	Text (categorical)	11 unique values: bakool, bay, galguduud, gado, hiran, lower juba, lower shabelle, middle juba, middle shabelle, mogadishu (banaadir), togdheer	Region in Somalia where the case was reported.
Reg.outbreak.no	Integer	≥1; blank if missing Range: 1 – 318 Unique values: 294	Regional outbreak number assigned during surveillance investigations.
National. outbreak.Number	Integer	≥1; blank if missing Range: 1 – 948 Unique values: 824	National outbreak number used to track linked outbreaks at the national level.
Adobe.page.ref	Integer	≥1	Page reference corresponding to the page number of the original scanned WHO line-list document from which the record was transcribed.
Died	Text (categorical)	yes, blank	Indicates whether the individual was identified as deceased from smallpox based on cross-referencing with WHO report. Blank indicates no death identified in available records.

Python software was used to perform selected data cleaning and comparison of the two independently transcribed datasets.

### Transcription of line-list variables and handling of missing data

From the original PDF document, data were transcribed for the following variables: *National Case Serial Number, Age, Sex, Date Onset Rash, Date Detected, Village/Locality, District, Region, Regional Outbreak Number, and ‘National Outbreak Number*.

The variables *Name* and *Specimen Result* were not transcribed. Case names were excluded to preserve data anonymity. The *Specimen Result* data was omitted due to inconsistent reporting formats in the original document, including entries such as “yes/no,” “number,” “date,” or missing information. This variability prevented consistent transcription.

A variable indicating *Death* was added to the transcribed dataset where this information could be identified through cross-referencing the line list records with WHO reports and outbreak documentations.

All data fields for each line list were transcribed, unless the information was unintelligible, not recorded or incomplete, in which case the cell was left blank. Where data were partially cut-off and not visible due to scanning errors of the physical document (as commonly occurred for *Reg Outbreak No* and/or *Nat Outbreak No*), the corresponding cells were also left blank. The percentages for missing values reported for each variable in the following section therefore includes missingness arising both from the original data collection and from transcription limitations due to illegible or incomplete source material.

For location data, where multiple spellings for a location name were identified, both variations were initially recorded and subsequently harmonized in accordance with historical maps.

### Recording national case serial numbers

**Fig. 4:**
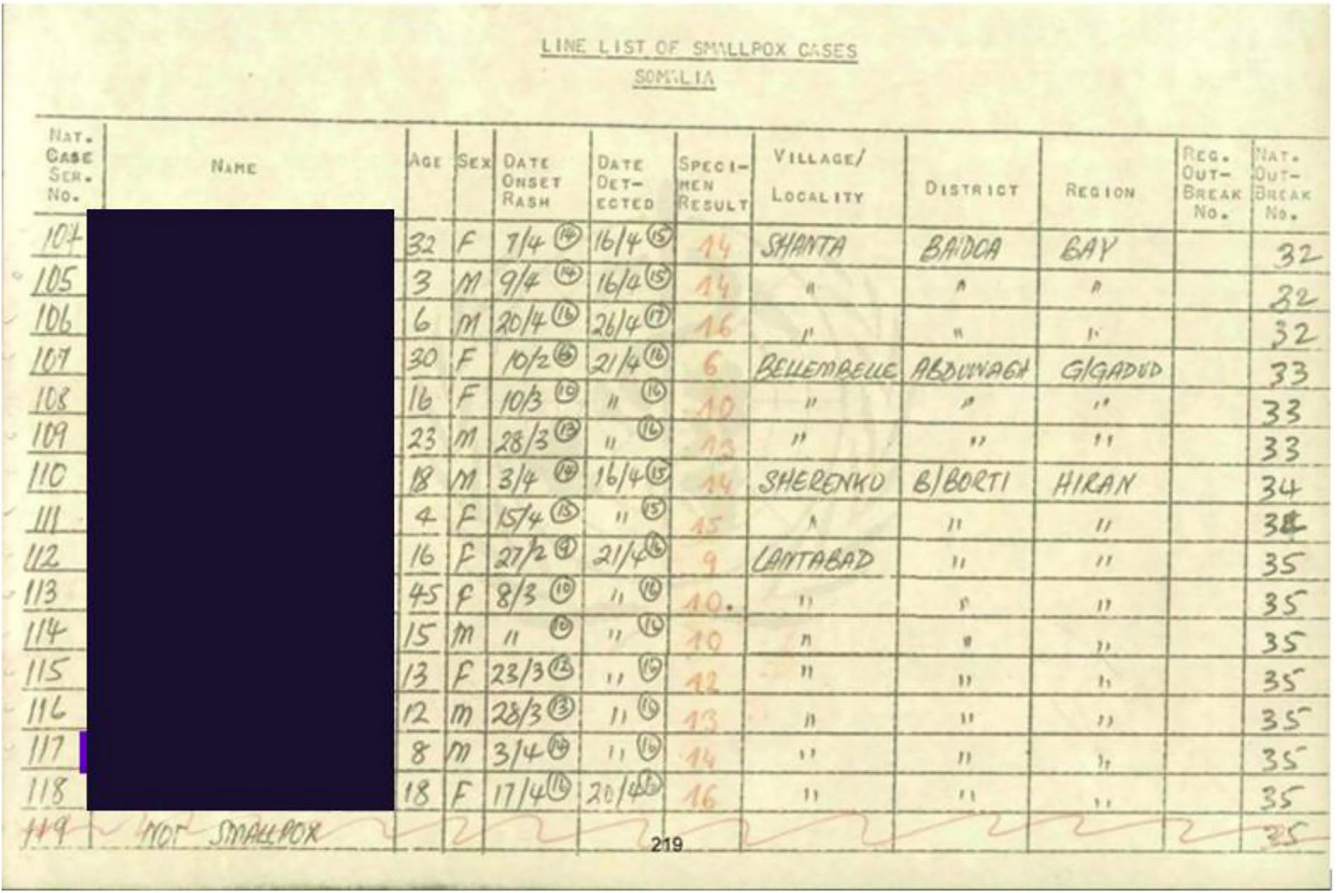
Screenshot of a national case serial number 119, marked as “NOT SMALLPOX”, World Health Organization 1976–1977.

**Fig. 5:**
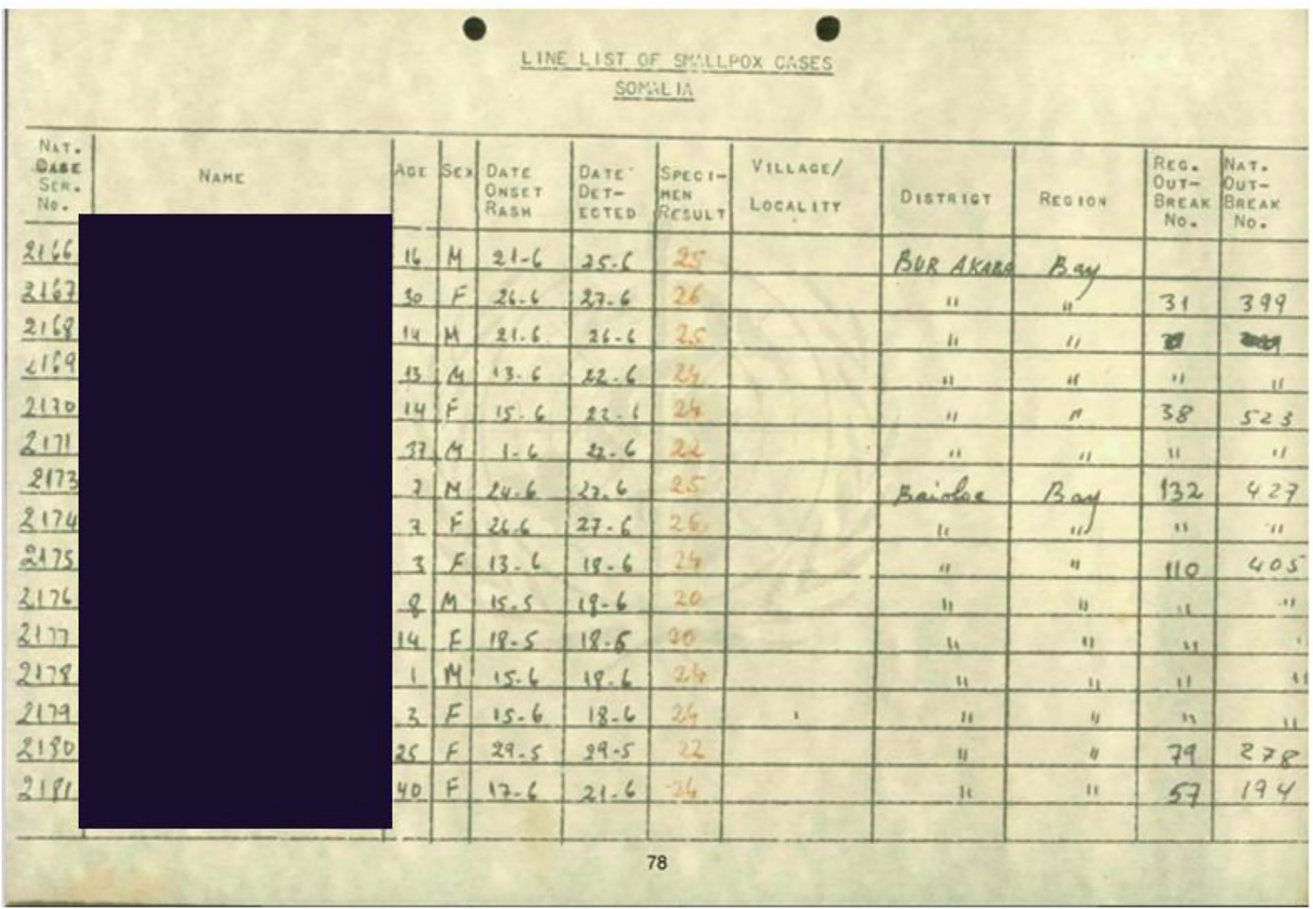
Screenshot showing a national case serial number skipped between 2171 and 2173, World Health Organization 1976–1977.

National case serial numbers were recorded directly from the PDF line list datasheets where available. Missing or inconsistent serial numbers were systematically reviewed. Initially, one serial number was crossed out in the original document, one was missing and skipped, and two cases lacked serial numbers but included all other relevant data. Specifically, the national case serial number 119 was crossed out in the line list document, identified as not being smallpox, and therefore excluded from transcription into the Excel datasheet (Fig. 4). Similarly, serial number 2172 was absent from the original PDF line list document and was not transcribed (Fig. 5).

**Fig. 6:**
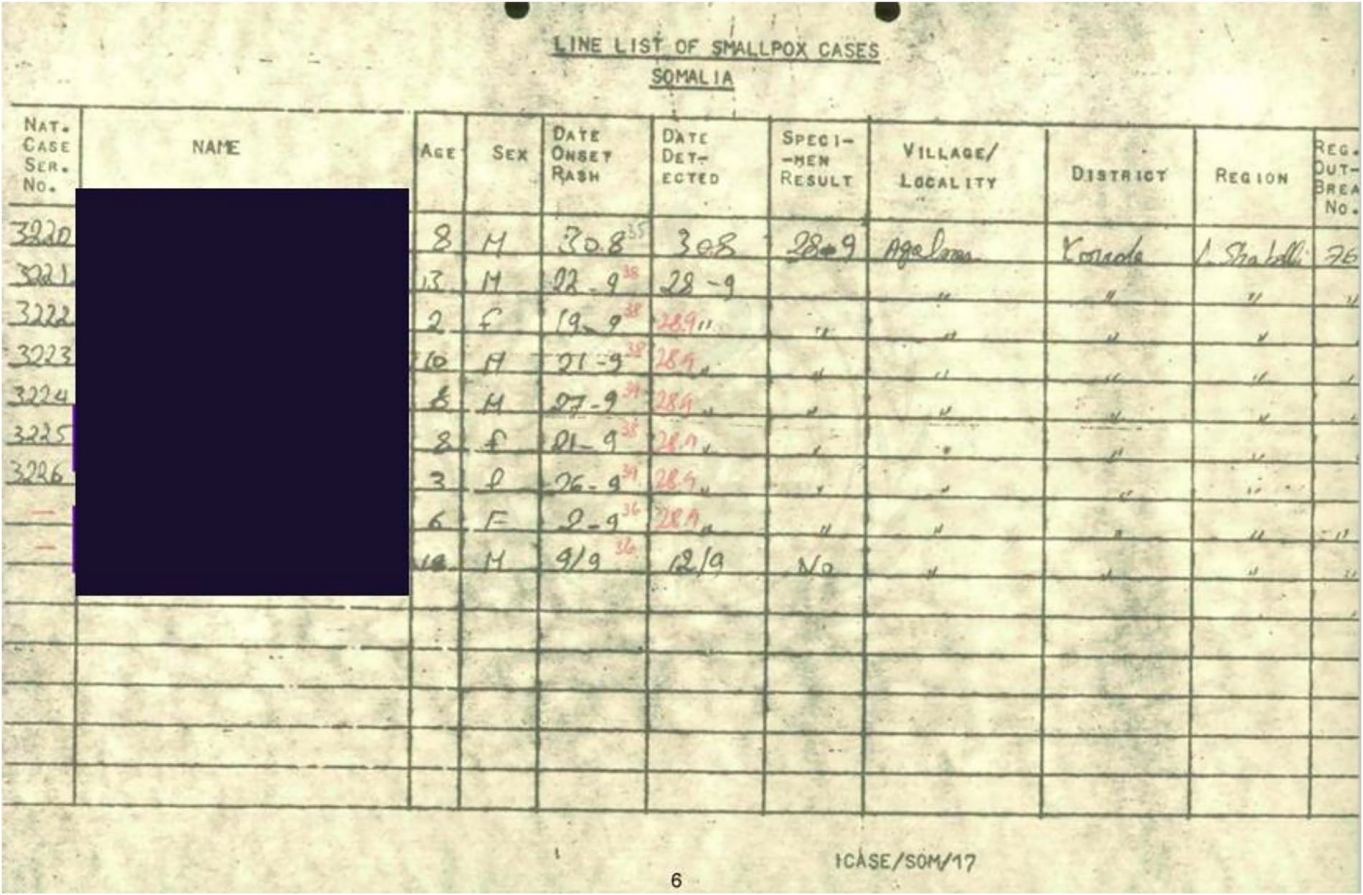
Screenshot of the last two smallpox cases without national case serial numbers, World Health Organization 1976–1977.

Two cases that initially lacked case serial numbers were subsequently assigned unique identifiers: 3227 and 3228 (Fig. 6). Case 3227 did not have a case serial number in both the *Line List of Smallpox Cases Somalia* and the *Outbreak Information* datasheets. Its number was inferred based on the village, district, region, and regional outbreak number from other cases reported on the same PDF page of the *Line List of Smallpox Cases Somalia* datasheet. Case 3228, which also lacked a case serial number in the *Line List of Smallpox Cases Somalia* datasheet, was assigned one from the *Outbreak Information* datasheet. The final dataset therefore includes national case serial numbers for all cases, resulting in 3,255 incident records of *variola minor* smallpox.

### Recording age

Ages were transcribed as recorded in the original *Line List of Smallpox Cases Somalia* datasheet. For individuals aged one year and above, ages were reported exclusively as whole numbers in years (e.g., 6, 21, 45), with no decimal or fractional values. These were transcribed directly without modification.

For infants under one year of age, age was recorded in months (e.g., “3 M” or “6 M”). To maintain a consistent age variable measured in years, these entries were transcribed as “0”. Age was missing in 139 records (4.3% of 3,255).

### Recording sex

Sex in the original line list datasheets was recorded as ‘m’ for male, ‘f’ for female, or was left blank. In the transcribed dataset, sex was similarly coded. Sex was missing in 121 records (3.7% of 3,255).

### Recording year for date of onset rash and date detected

The variable *Date Detected* refers to the date on which WHO authorities, or the surveillance teams under WHO coordination, first identified and recorded a case presenting with rash and fever. It does not represent the date of symptom onset or laboratory confirmation.

In the original document, the majority of dates were recorded as ‘day/month’ only, without specifying the year. In such cases, the year was standardised as 1977 in the transcribed dataset. This decision was based on epidemiological context as only 53 global smallpox cases were reported in 1976, of which 39 occurred in Somalia prior to January 1977, while the majority of remaining cases occurred in 1977^9^. As Somalia represented the final reservoir of smallpox transmission and most outbreak activity occurred in 1977, this was considered the most plausible year for records lacking explicit year information.

Where the year was explicitly recorded in the original line list, it was transcribed as documented. All dates are presented in dd/mm/yyyy format. One entry listed a detection date of 31/06, which was corrected to 30/06 to reflect the calendar month of June.

Date of Onset Rash was missing in 44 records (1.4% of 3,255), and Date Detected was missing in 131 records (4.0% of 3,255).

### Recording death

The original linelist PDF datasheets did not include information on smallpox associated deaths. To identify fatalities among individuals listed in the line lists, the 1979 WHO report *Smallpox Eradication in Somalia* was consulted^4^. The report includes a table titled *Reported Smallpox Deaths, Somalia 1977* (Table 4.10), which provides information on the region, outbreak number, initials, sex, age, vaccination scar status, and the dates of rash onset, detection, and death for 12 deceased individuals^4^. Records for the deceased individuals were cross-referenced with the PDF line list datasheets, and a *death* column was subsequently added to the final Excel dataset.

Six deaths were matched to individuals in the line lists, corresponding to national case serial numbers 167, 513, 1021, 1967, 2860, and 3243. However, the remaining six deaths could not be matched due to missing identifier for variables such as name, sex, age, or national outbreak numbers in the PDF line list. One additional death (national case serial number 3073) was identified in an *Outbreak Information* datasheet, that was not reported in the WHO Smallpox Eradication in Somalia report. In total, seven deaths are reported in the transcribed dataset.

### Recording geographical data

The line list PDF document contained geographic information split into three administrative levels: *Region, District* and *Village*.

To maintain consistency with the WHO report *Smallpox and Its Eradication (Fenner et al*., *1988)*, the transcribed data were updated such that the names of the districts and regions matched that of the report, despite frequent spelling inconsistencies in the original PDF document^1^. One entry in the original line list document referred to the district Genale which is not listed as a district in the WHO Report. Further investigation showed that Genale is a town located in the Merca district, and the dataset was updated to reflect this.

Geolocation of villages was achieved through reference to a Somalia settlement dataset made available through the United Nations Office for the Coordination of Humanitarian Affairs (OCHA). This dataset, which is available on https://data.humdata.org/dataset/somalia-settlements-p-coded-shapefile, contains geographic locations of over 10,000 settlements in Somalia, with information sourced from surveys completed by the United Nations Development Programme (UNDP) in both 1997 and 2006, the Food Security Assessment Unit (FSAU) and the Kenya Medical Research Institute (KEMRI).

The locations of 467 villages were identified from the OCHA dataset. Exact matches between the datasets were uncommon, and therefore each located village was assigned a confidence rating of either ‘good’, ‘moderate’ or ‘poor’. A ‘good’ rating indicates that the village names either matched, or that any inconsistencies followed the patterns of common transliterations (‘h’ for ‘x’, ‘q’ for ‘k’, etc.), or common misinterpretation of the original PDF (‘d’ for ‘ol’, ‘n’ for ‘r’, etc.). A ‘moderate’ rating indicates similar spellings that do not follow common transliterations. A ‘poor’ rating indicates further spelling discrepancies, or the presence of multiple similarly named places.

The original village names were paired with the corresponding names in the OCHA dataset (including the alternate spellings where available), along with the associated decimal longitude and latitude coordinates, and the data sources as provided by the OCHA dataset. Where appropriate a brief justification of the assigned confidence rating was also provided.

Information on Region, District, and Village were missing in 23, 60, and 207 records, respectively (0.7%, 1.8% and 6.4% of 3,255 records).

### Regional and national outbreak number

*Regional Outbreak Number* and *National Outbreak Number* were transcribed as integers in the Excel dataset. Due to scanning inconsistencies of the original hard-copy document, the columns for Regional Outbreak Number and National Outbreak Number were occasionally cut off and not visible in the PDF. As a result, a large number of corresponding cells for these two variables were left blank. Where possible, missing data were supplemented by cross-referencing with the *Outbreak Information* datasheets. Missing values were more common for the Regional Outbreak Number than for the National Outbreak Number, with 1,069 and 514 missing records, respectively (32.8% and 15.8% of 3,255 records).

### Adobe PDF page number

The Adobe PDF page number of the original scanned document was recorded in the transcribed dataset to enable the independent verification of entries and facilitate cross-checking of transcribed records against the source document.

## Data Records

The dataset is available at UK Data Service ReShare Repository as a compressed (.zip) folder titled “857734_bundle”, which is the identifier assigned by the repository^10^. The compressed folder contains two files:

1. **“2025_03_03_Read Me Document_Transcribed Smallpx Dataset_Reshare Repository.docx”**: A Microsoft Word document providing descriptive documentation for the dataset, including project title, dataset scope, and a summary of the variables included in the transcribed line list.
2. **“2025_03-03 Somalia Data Entry v1.0.xlsx”**: A Microsoft Excel file containing the transcribed smallpox line list dataset. The dataset comprises 3,255 records of individual *variola minor* smallpox cases in Somalia.

## Technical Validation

### Data validation and determination of transcription error rate

Minor data cleaning and processing were undertaken during transcription to ensure the transcribed dataset reflected the original PDF document as accurately as possible. Each round of data entry was followed by a structured validation process conducted by independent researchers to assess transcription accuracy.

Following the first round of data entry, approximately 10% random sample of the transcribed line list cases (i.e. about 327 cases) were manually reviewed and cross-referenced with the original PDF document to estimate the data transcription error rate (%). The data discrepancies were manually corrected.

After the second round of transcription, the two independent datasets were compared using a Python script to identify discrepancies. For each discrepancy, team members referred to the original PDF document to determine the correct entry. The first transcription was designated as the master dataset and updated with the corrected entries. With this error correction process completed, a random sample of 20% of line list cases (651 cases) was drawn from the master dataset and compared to the original PDF document to determine a final transcription error rate.

The initial data validation identified a transcription error rate of 1.60% (51 errors in 327 cases, with 10 data points per line). After the second validation round, a final error rate of 1.01% (Table 3) was reported.

**Table 3:** Transcription error rates by variable and overall dataset error rate.

Variable	Age	Sex	Date onset	Date detected	Village	District	Region	Regional outbreak number	National outbreak number	Total
Transcription error rate	0.46%	0.15%	1.08%	0.46%	1.23%	0.46%	0.46%	1.84%	2.92%	1.01%

Previous studies reporting transcription of historical datasets suggests acceptable error rates ranging between 0.1–0.5%^11,12^. The slightly higher error rates observed in this dataset are largely attributable to difficulties transcribing regional and national outbreak numbers, where portions of the original documents were truncated during the scanning process.

### Limitations and challenges of the transcribed dataset

The overall level of smallpox case ascertainment cannot be formally quantified because the results of laboratory specimens collected to confirm suspected cases are not available in the original line list datasheets. As a result, the dataset primarily represents cases identified based on clinical symptoms. Without access to laboratory confirmation results, it is not possible to determine the proportion of clinically diagnosed cases that were subsequently confirmed as smallpox or to estimate the sensitivity of the surveillance system.

Details regarding the rules and procedures used for data collection and entry into the line list documents in Somalia during 1976–1977 remain unavailable, as no standard operating procedures (SOPs) used by the data officers at the time could be located.

A major limitation of the dataset is that blank cells may reflect either non-recording during the original outbreak investigation or illegibility at the time of transcription. These mechanisms may introduce reporting or information bias in the former case and measurement or transcription bias in the latter, potentially resulting in non-random patterns of missingness that could influence downstream analyses.

Ages recorded in the dataset represent reported values rather than ages derived from verified dates of birth. Given that many affected populations were nomadic, some degree of recall bias and age rounding may be present, which could introduce uncertainty in age-specific analyses.

For records in which the original line list reported only the day and month, the year was standardised to 1977. As explicitly recorded and inferred years cannot be distinguished in the transcribed dataset, this may introduce minor uncertainty in analyses requiring precise year-level temporal resolution.

Regarding the transcription of village names, although efforts were made to accurately transcribe the names and reconcile them with historical maps, the large number of village entries created difficulties in interpreting handwriting, and variations in spelling further complicated this process. Due to their reduced granularity at the level of village name, transcriptions of district and regional information are deemed more accurate.

Changes in surveillance intensity over the course of the outbreaks, including the introduction of a cash reward scheme in February 1977, may have influenced case identification and reporting patterns in the subsequent period, and should therefore be considered when interpreting temporal trends in the dataset.

## Usage Notes

The transcribed dataset is provided in a Microsoft Excel format (.xlsx) which is compatible with most statistical software packages and general-purpose programming languages. This enables the dataset to be readily used for descriptive epidemiological analyses and to be integrated with the 1975 Somalia census data for broader studies of smallpox transmission and outbreak dynamics.

## Data Availability

The dataset is available for download from the UK Data Service ReShare Repository under the title *Transcribed Historic Data from the Last Recorded Smallpox Outbreaks in Somalia, 1976–1977*^10^. The dataset can be accessed at: https://reshare.ukdataservice.ac.uk/857734/. The data are openly available and can be downloaded and used without restriction or prior permission.

## Code availability

For transparency purposes, the Python code used for data validation and the Jupyter notebook Date_entry_check.ipynb is uploaded to GitHub on the publicly available repo SmallpoxSomaliaReleased.git.
